# The relationship between perceived social support with anxiety, depression, and insomnia among Chinese college students during the COVID-19 pandemic: The mediating role of self-control

**DOI:** 10.3389/fpsyt.2022.994376

**Published:** 2022-10-06

**Authors:** Guojun Zhao, Fusen Xie, Shuangchen Li, Yuee Ding, Xu Li, Haijian Liu

**Affiliations:** ^1^School of Psychology, Northwest Normal University, Lanzhou, China; ^2^Academy of Plateau Science and Sustainability, Qinghai Normal University, Xining, China; ^3^Mental Health education Center, Gansu Health Vocational College, Lanzhou, China; ^4^School of Education, Northwest Normal University, Lanzhou, China

**Keywords:** COVID-19, college students, perceived social support, self-control, mental health

## Abstract

**Object:**

We aimed to investigate the associations between perceived social support and anxiety, depression, and sleep disturbance *via* self-control among Chinese college students during the COVID-19 pandemic.

**Materials and methods:**

The Perceived Social Support Scale, Self-control Scale, Self-rating Anxiety Scale, Self-rating Depression Scale, and Insomnia Severity Index Scale were used to survey 1,997 college students during the COVID-19 pandemic, who submitted valid questionnaires (*M*_*age*_ = 19.93, *SD*_*age*_ = 1.47, Range = 18–24 years, 62% female).

**Results:**

The perceived social support and self-control were significantly positively correlated, and they were significantly and negatively associated with anxiety, depression, and insomnia. Further analysis found that self-control partially mediated the relationships between perceived social support with anxiety, depression, and insomnia.

**Conclusion:**

During the COVID-19 pandemic, Chinese college students’ self-control played a partial mediating effect in the relationships between perceived social support and anxiety, depression, and insomnia. This study provides new insights and inspiration for improving college students’ mental health in the context of the pandemic.

## Introduction

The COVID-19 pandemic has been spreading worldwide since its outbreak in December 2019. Up till now, many countries have been experiencing the second or third wave of the COVID-19 pandemic. The repeated and constant pandemic has greatly disturbed people’s daily life and threatened their physical and mental health (1). For college students, they have to change their lifestyles and reduce their social activities during the COVID-19 pandemic (2). In the context of social isolation, the reason for the limitations of their living environment, and the pressure from online learning, they are more likely to suffer from mental health problems (3, 4), including anxiety, depression, and insomnia. Zhang et al. (5) reported that the prevalence rates of anxiety and depression among Chinese college students were 10.3 and 25.1% during the COVID-19 pandemic, respectively. Additionally, research evidence showed that 16.9% of students once suffered insomnia in varying degrees during the pandemic (6). Therefore, it is necessary and urgent to detect the college students’ mental health hit by the COVID-19 pandemic and help them effectively alleviate anxiety, depression, and insomnia.

Perceived social support refers to individuals’ subjective feelings and judgments of the support from society, which includes material support, psychosocial support, and emotional guidance (7). Some studies have certified the association between perceived social support and mental health from both theoretical and practical aspects. As an important external factor, social support is beneficial to individual’s mental health and can alleviate their anxiety, depression, and sleep disturbance (8, 9). Moreover, some studies have proved that social support plays a positive role in predicting individuals’ mental health during the COVID-19 pandemic (10–12). Individuals with higher levels of perceived social support face fewer mental health problems, such as anxiety, depression, and insomnia (11, 12). Therefore, in this study, we speculated that the perceived social support of Chinese college students was negatively correlated with anxiety, depression, and insomnia during the COVID-19 pandemic.

Self-control refers to the ability of individuals to regulate, manage, and control their impulsive ideas, emotions, and behaviors (13). It is helpful in better regulating individuals’ behaviors and emotions and guiding them in making rational decisions between desired goals and pleasurable responses (13–15). A large body of evidence has shown the importance of self-control for people’s life adaptation. For example, individuals with higher levels of self-control generally reported higher levels of subjective wellbeing and a better state of health (16, 17). Self-control, as an internal factor, is closely related to individual’s mental health. Individuals with higher levels of self-control tend to have greater perseverance and better emotional regulation, and thus are able to adapt more successfully to different situations and ultimately have the opportunity to experience more positive emotions (18, 19). In contrast, previous studies have demonstrated that self-control has negative associations with anxiety and depression (20, 21). In addition, self-control is regarded as an important internal factor that influences individuals’ sleep. Self-control can help individuals resist various disturbances from the external environment and cultivate sound sleep habits and improve sleep quality (22).

Furthermore, there is a close link between perceived social support and self-control. Social support, as an external factor, can supply the cognitive resources needed for self-control, which in turn increases the level of self-control (23). The past study provided evidence that people would show high levels of self-control when they experience more social support among Chinese college students (24). Moreover, the buffering effect model of social support proposes that social support can buffer the effect of negative life events on individuals based on the internal cognitive system, thus promoting individuals’ mental health (25). In this process, self-control is an important factor supporting the cognitive system, which can restrain impulsive responses and ensure that the cognitive process complies with intended goals (13).

Some studies have found that self-control can mediate the association between social support and life adaptation, such as the indirect predictions of social support on problem behavior and subjective wellbeing (24, 26). As mentioned earlier, both perceived social support and self-control are closely related to individual’s mental health, and social support can promote individual’s ability of self-control. Therefore, we speculated that self-control might play a mediating role in the relationships between social support and anxiety, depression, and insomnia of college students during the COVID-19 pandemic.

Although many studies have discussed the association between perceived social support and mental health, there is still a lack of study on the underlying mechanisms of the relationships between social support and anxiety, depression, and insomnia among college students during the COVID-19 pandemic. As a kind of external positive resource, social support can directly provide emotional support, understanding, and encouragement to college students during the COVID-19 pandemic, thereby alleviating their negative mental states, such as anxiety, depression, and insomnia, and also enhancing their self-control, which in turn can improve emotional wellbeing and sleep quality. Due to the outbreak of the COVID-19 pandemic, college students have to change their learning styles and living habits. Various coping measures preventing the novel coronavirus, such as social isolation, online learning, and increased time alone, make it more likely for college students to have mental health problems, which may require more social support and objectively challenge their self-control.

**FIGURE 1:**
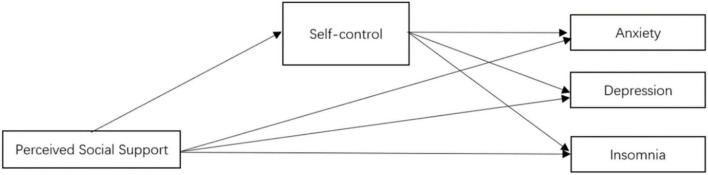
The conceptual model, social support as the independent variable, self-control as the mediating variable, anxiety, depression, and insomnia as the dependent variables.

Therefore, this study aimed to examine the prediction of social support for mental health *via* self-control among Chinese college students during the COVID-19 pandemic. Perceived social support might directly predict anxiety, depression, and sleep insomnia during the pandemic. Furthermore, perceived social support might also indirectly predict anxiety, depression, and insomnia *via* the mediating role of self-control. The hypothesis model of this study is shown in Figure 1.

## Materials and methods

### Participants

G*Power version 3.1.9.7 was used to conduct an *a priori* power analysis calculation for the adequacy of sample size (27). This result suggested that detecting a medium effect size (*D* = 0.3, α = 0.05) with a power (1 − β) of 99% needed a sample size of 188. This research was conducted in seven colleges and universities in Lanzhou City, the capital of Gansu Province, in China on October 2021. During the period, China was experiencing the second wave of the COVID-19 pandemic, and people infected with the Novel Coronavirus had been detected in Lanzhou for the first time. All colleges and universities in Lanzhou had taken emergency measures against the pandemic, with students being quarantined on campus and taking online learning. In terms of sampling principal in our study, we aimed to explore the impact of social environment and atmosphere under the pandemic on mental health instead of the direct effect of being infected by the virus. Therefore, we made three criteria to recruit the participants of college students: first, the colleges and universities had been identified as in severity level of the COVID-19 pandemic, students were not allowed in close contact with others, and limited the scope of activity in the period of the pandemic. Second, the students themselves and their close friends and family members, were not infected by the virus. Third, the students had no physical illnesses or psychological problems that appeared before the COVID-19 outbreak. We adopted the convenience sampling method to collect information *via Questionnaire Star* which is a popular online survey platform in China. The study design was approved by the ethics review board of Northwest Normal University. All participants were asked to provide informed consent and completed all questionnaires anonymously in the period between 15 and 22 October 2021. And an online shopping coupon worth 2 Yuan was provided to each of the participants for thanking them for answering the questionnaires. A total of 2,369 college students took part in the survey. However, 372 college students were excluded from the analysis because they did not fill in the informed consent form, did not complete all items of the questionnaires, or did not meet the recruiting criteria. Finally, a total of 1,997 valid questionnaires provided by the participants (*M*_*age*_ = 19.93, *SD* = 1.47, Range = 18–24 years old, 62% female) were included in the analysis.

### Measures

#### Perceived social support

The Chinese version of the Perceived Social Support Scale (PSSS) was used to evaluate perceived social support (28). The scale contains 12 items, and each item is rated on a 7-point Likert scale (“1 = complete disagree”; “7 = complete agree”). A higher total score on the scale indicates that an individual can perceive more social support. The previous study has proven the validity and reliability of this scale in China (10). In this study, the Cronbach’s α coefficient was 0.95.

#### Self-control

Self-control was evaluated with the Chinese version of Tangney and colleagues’ Self-Control Scale (SCS) (29). The scale contains 19 items, and each item is rated on a 5-point Likert scale (“1 = strongly disagree”; “5 = strongly agree”). Fifteen items are reversed so that a higher total score indicates stronger self-control. This scale has been used in Chinese college students, showing good reliability and validity (30). In this study, the Cronbach’s α coefficient of this scale was 0.89.

#### Anxiety

The Self-Rating Anxiety Scale (SAS) was used to evaluate the participants’ anxiety symptoms (31). The scale has 20 items, rated on a 4-point Likert scale (“1 = rarely”; “4 = always”). Five items were reversed so that the higher total score on the scale indicated more severe symptoms. The scale has been used by Chinese college students and showed good reliability and validity (32). In this study, the Cronbach’s α coefficient of this scale was 0.76. In addition, the standard of the severity of anxiety can be assessed by transforming it into an index score by multiplying the sum of the raw score of 1.25. The index score has two types: no anxiety (<50) and anxiety (≥50).

#### Depression

The SAS was used to assess the participants’ symptoms of depression (33). This scale contains 20 items (10 positive and 10 negative). Each item is rated on a 4-point Likert scale (“1 = rarely”; “4 = always”). After the positive items were reversed, the higher total score on the scale indicated more severe symptoms. This scale has been used in Chinese college students and demonstrated good reliability and validity (32). The Cronbach’s α coefficient of this scale in this study was 0.86. The standard of the severity of depression can be assessed by transformation to an index score by multiplying the sum of the raw score of 1.25, and divided into two types: no depression (<53) and depression (≥53).

#### Insomnia

The Insomnia Severity Index Scale (ISI) was used to assess the severity of insomnia (34). The scale contains seven items, rated on a 5-point Likert scale (“1 = rarely”; “4 = always”). A total score ranges between 0 and 28, with higher total scores indicating more severe insomnia, where 0–7 indicates the absence of insomnia, 8–14 subthreshold insomnia, 15–21 moderate insomnia, and 22–28 severe insomnia. This scale has been used in Chinese college students, showing good reliability and validity (35). In this study, the Cronbach’s α coefficient of this scale was 0.87.

### Data analysis

SPSS 24.0 was used to deal with the data analysis. First, a common method bias was tested by the Harman single test. Second, descriptive statistics and correlation analyses were conducted for exploring the relationships between variables in this study. An independent samples *t*-test was conducted to check the gender differences between variables. Finally, Model 4 of the PROCESS 3.4 was used to examine the mediating effects of self-control on the relationships between perceived social support, anxiety, depression, and insomnia. The test of mediating effect was based on a linear regression model and bootstrap analysis of 5,000 samples. Effect estimates and bias-corrected 95% confidence intervals (BC 95% CIs) were obtained.

## Results

### Common method bias test

A common method bias is likely to occur due to using a series of self-report questionnaires to collect data (36). Therefore, Harman’s one-factor test was performed and showed 12 factors with the eigenvalues above one. The analysis showed that the first factors presented 20.66% of the variance, which did not reach the criterion of 40%. Therefore, there was no serious common method bias in this study.

### Descriptive and correlation analysis

According to the diagnostic index scores, the prevalence rates of anxiety and depression among participants in this study were 12.61% (252/1997) and 29.89% (597/1997), respectively. And a total of 29.64% (592/1997) of participants had moderate insomnia and 8.51% (170/1997) had severe insomnia.

Means, standard deviations, and Pearson correlation coefficients of the variables were shown in Table 1. The result suggested that perceived social support had significant and negative correlations with anxiety, depression, and insomnia. Self-control was significantly and negatively related to anxiety, depression, and insomnia. Moreover, perceived social support was significantly and positively related to self-control. Additionally, the results revealed that age was significantly correlated with self-control, anxiety, depression, and insomnia.

**TABLE 1:** Descriptive statistics and correlations of perceived social support, anxiety, depression, and insomnia (*N* = 1,997).

Variable	*M (SD)*	1	2	3	4	5	6	7
(1) Gender	–	−						
(2) Age	–	–0.034	−					
(3) Perceived social support	4.87 (1.08)	0.042	0.062	−				
(4) Self-control	3.40 (0.56)	–0.017	0.116**	0.364**	−			
(5) Anxiety	1.65 (0.30)	0.118**	−0.089**	−0.371**	−0.408**	−		
(6) Depression	1.89 (0.42)	0.077**	−0.088**	−0.532**	−0.468**	0.716**	−	
(7) Insomnia	1.92 (0.79)	−0.045*	−0.051*	−0.286**	−0.360**	0.487**	0.429**	−

*p < 0.05, **p < 0.01.

An independent samples *t*-test was conducted to examine the gender differences in research variables. The results revealed that gender differences were significant in anxiety, depression, and insomnia. Female students reported higher scores in anxiety (*t* = −5.313, *p* < 0.001) and depression (*t* = −3.448, *p* < 0.01) compared to their male peers, while lower score in insomnia (*t* = 2.024, *p* < 0.05). Additionally, the gender differences were not significant in perceived social support (*t* = 0.768, *p* = 0.443) and self-control (*t* = −1.875, *p* = 0.061).

### The mediating effect of self-control

**FIGURE 2:**
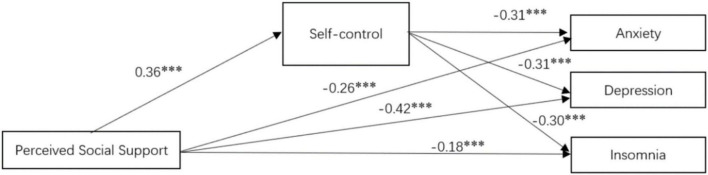
The integrated mediation model. ^***^p < 0.001.

This study further detected whether self-control mediated the relationships between perceived social support and anxiety, depression, and insomnia. Model 4 of PROCESS 3.4 (37) was used to perform three mediation analyses with gender and age as the controlled variables, perceived social support as the independent variable, self-control as the mediating variable, and anxiety, depression, and sleep insomnia as the dependent variables, respectively. The integrated mediation model was shown in Figure 2. Perceived social support positively predicted self-control (β = 0.36, *p* < 0.001) and negatively predicted anxiety (β = −0.26, *p* < 0.001), depression (β = −0.42, *p* < 0.001), and insomnia (β = −0.18, *p* < 0.001), respectively. Moreover, self-control negatively predicted anxiety (β = −0.31, *p* < 0.001), depression (β = −0.31, *p* < 0.001), and insomnia (β = −0.30, *p* < 0.001), respectively.

First, when anxiety was the dependent variable in the model, the total effect of perceived social support on anxiety was −0.3725 (LLCI = −0.1140, ULCI = −0.0917). The direct effect was significant, with the effect size being −0.2622 (LLCI = −0.0838, ULCI = −0.0610). The meditating effect on self-control was significant, with the size of meditating effect of −0.1103 (LLCI = −0.0366, ULCI = −0.0248), accounting for 29.61% of the total effect of perceived social support on anxiety, as shown in Table 2.

**TABLE 2:** The mediating role of self-control in the relationship between perceived social support and anxiety.

Effect type	Effect size	Boot SE	95% CI		Proportion of effect size
Total effect	−0.3725	0.0057	−0.1140	−0.0917	
Direct effect	−0.2622	0.0058	−0.0838	−0.0610	70.39%
Indirect effect	−0.1103	0.0030	−0.0366	−0.0248	29.61%

Second, when depression was the dependent variable in the model, the total effect of perceived social support on depression was −0.5325 (LLCI = −0.2220, ULCI = −0.1932). The direct effect was significant, with the effect size −0.4209 (LLCI = −0.1786, ULCI = −0.1495). The meditating effect on self-control was significant, and the size of the meditating effect was −0.1116 (LLCI = −0.0523, ULCI = −0.0353), accounting for 20.96% of the total effect of perceived social support on depression. As shown in Table 3.

**TABLE 3:** The mediating role of self-control in the relationship between perceived social support and depression.

Effect type	Effect size	Boot SE	95% CI		Proportion of effect size
Total effect	−0.5325	0.0074	−0.2220	−0.1932	
Direct effect	−0.4209	0.0074	−0.1786	−0.1495	79.04%
Indirect effect	−0.1116	0.0043	−0.0523	−0.0353	20.96%

Finally, when insomnia was the dependent variable in the model, the total effect of perceived social support on insomnia was −0.2820 (LLCI = −0.2365, ULCI = −0.1750). The direct effect was significant, with the effect size −0.1756 (LLCI = −0.1598, ULCI = −0.0965). The meditating effect of self-control was significant. The size of the meditating effect was −0.1064 (LLCI = −0.0951, ULCI = −0.0617), accounting for 37.73% of the total effect of perceived social support on insomnia, as shown in Table 4.

**TABLE 4:** The mediating role of self-control in the relationship between perceived social support and insomnia.

Effect type	Effect size	Boot SE	95% CI		Proportion of effect size
Total effect	−0.2820	0.0157	−0.2365	−0.1750	
Direct effect	−0.1756	0.0161	−0.1598	−0.0965	62.27%
Indirect effect	−0.1064	0.0086	−0.0951	−0.0617	37.73%

All in all, the results showed that self-control played a partially mediating effect in the relationships between perceived social support and anxiety, depression, and insomnia. Therefore, our hypothesis was supported.

## Discussion

This study examined the relationships between perceived social support and anxiety, depression, and insomnia, especially the mediating effects of self-control among Chinese college students during the COVID-19 pandemic. We found that the prevalence rates of anxiety and depression were 12.61 and 29.89%, and of moderate and severe insomnia were 29.64 and 8.51%, respectively. All the results are higher than the prevalence of non-pandemic situations, which indicates that the pandemic has caused more serious mental health problems (32, 35). The long-term lasting of the COVID-19 pandemic has disturbed the normal life and study style of college students. Offline classes were switched to online learning in dormitories to avoid face-to-face contact between teachers and students and between students, so as to cut down the spread and infection of the pandemic. This situation is likely to adversely affect college students’ mental health and cause them to suffer from anxiety, depression, and insomnia (4, 38, 39).

We found that perceived social support had a significantly positive correlation with self-control while significant negative correlations with anxiety, depression, and insomnia. These results supported our hypotheses. Our findings are consistent with prior studies (8, 9). Perceived social support of college students during the COVID-19 pandemic can significantly and negatively predict their anxiety, depression, and insomnia. Due to suffering more pressure of study and living during the COVID-19 pandemic, college students urgently needed the material and emotional supports provided by significant others to help them overcome these difficulties. If they could perceive adequate and effective support, their negative mental health would be improved. The results showed that college students with greater perceived social support usually suffered less from anxiety, depression, and insomnia during the COVID-19 pandemic. It reminds that college students should actively acquire and perceive available social resources to effectively prevent and alleviate their mental health problems arising during the COVID-19 pandemic.

Furthermore, the results also showed that self-control could play a partial mediating role in the relationships between social support and anxiety, depression, and insomnia of college students. Previous studies indicated that individuals’ ability to self-control significantly and negatively predicted anxiety, depression, and sleep disturbance (20–22). During the COVID-19 pandemic, maintaining the normal style of study and living has been challenged. In this case, college students with stronger self-control are more possible to keep healthy living habits, better regulate their emotions, and experience more happiness, than buffering anxiety and depression caused during the COVID-19 pandemic (19, 40). In addition, better self-control ability can also help college students keep good sleep habits during the pandemic and avoid sleep-related problems (41). On the other hand, previous research has provided evidence that social support can positively predict the self-control of college students (23). When various social supports from significant others are perceived by college students, it is likely to enhance their sense of control over their life and study, and thus improve their self-control. In line with the energy model of self-control, social support is beneficial for college students to compensate for limited cognitive resources consumed by self-control during the COVID-19 pandemic (23) so that they can better restrain their impulsive behaviors and irrational ideas related to mental health problems. Therefore, perceived social support is not only directly associated with college students’ anxiety, depression, and insomnia during the COVID-19 pandemic but also indirectly connected with these variables of mental health *via* self-control. It suggests that college students can maintain and promote their mental health by actively perceiving external social support and improving internal self-control.

The results of this study have significant implications for preventing the risk of psychological problems. When providing support for vulnerable people such as college students, self-control should be fully considered. Students should be encouraged to actively use their psychological strength to solve their problems instead of passively waiting for support from others and universities. Self-control as a powerful adaptive ability can be improved through constant practice or promoted by cognitive adjustment, which cannot only directly help students overcome difficulties but also indirectly transmit the beneficial effect of social support to students in trouble. Therefore, healthcare providers and researchers should consider how to effectively integrate both self-control of internal resources and social support of external resources into a set of comprehensive counseling strategies for the mental health of college students in the context of unexpected social events. And it also suggests that future studies on mental health during the COVID-19 epidemic should not only examine the independent influence of external factors or internal factors but also pay more attention to the possible link between internal and external factors.

## Limitations and future research

First, all data of this study were obtained based on the psychometric model. The scale scores reflect the general characteristics and trends of the sample group, but lack attention to special phenomena, and cannot present complete and detailed individual information. Some case studies and in-depth interviews may be used to outline a deep and specific psychological state and behaviors related to our research variables in the pandemic. Future study is suggested to consider the combination of measurement, case study, and in-depth interview method to explore the impact of the COVID-19 pandemic on college student’s mental health.

Second, we did not collect more demographic information in this study. To respect personal privacy and relieve the participants’ burden and worry of answering the questionnaires, we advised the participants to respond to the information about the demographic characteristics to their own will, but gender and age were required. When we analyzed the data, we found that few participants filled all optional items, so we only reported their gender and age. However, family background and socioeconomic status have potential effects on research variables, especially for social support. Therefore, we suggest that future researchers should measure these demographic variables.

Third, although a large number of cross-sectional data have been collected, our study only provided evidence of correlation rather than causal relationship when exploring the potential risk of the COVID-19 pandemic. We did not conduct follow-up studies and did not collect retrospective data, so this study could not comprehensively reveal the psychological changes of college students. Future research is expected to investigate the effect of the pandemic on mental health using experimental design or longitudinal study design.

Finally, although this study examined the inverse influence of the COVID-19 pandemic, we should realize that people in difficulty have been attempting to adapt to such dramatic social change. The vast majority of studies have been focusing on the dark side, ignoring the positive strength of human beings. Although we had emphasized the role of self-control, we did not connect it with other positive variables, such as happiness. Therefore, future research should pay more attention to positive psychological factors and explore how people actively adjust themselves to the stress caused by major social events.

## Conclusion

During the COVID-19 pandemic, Chines college students’ mental health is associated with the social support of external factors and self-control of internal factors, and self-control mediates the relationship between social support and mental health. It implies that the internal positive force of an individual is beneficial to transmit the positive effect of external support on health to individuals.

## Data availability statement

The raw data supporting the conclusions of this article will be made available by the authors, without undue reservation.

## Ethics statement

The studies involving human participants were reviewed and approved by the Research Ethics Commission of the School of Psychology, Northwest Normal University. The patients/participants provided their written informed consent to participate in this study.

## Author contributions

GZ and FX designed this study and wrote the manuscript. SL, YD, and XL recruited the participants and collected the data. FX performed the data analyses. All authors contributed to the study conception, design, read, and approved the final manuscript.
